# MoCA vs. MMSE of Fibromyalgia Patients: The Possible Role of Dual-Task Tests in Detecting Cognitive Impairment

**DOI:** 10.3390/jcm10010125

**Published:** 2021-01-01

**Authors:** Alvaro Murillo-Garcia, Juan Luis Leon-Llamas, Santos Villafaina, Paloma Rohlfs-Dominguez, Narcis Gusi

**Affiliations:** 1 Physical Activity and Quality of Life Research Group (AFYCAV), Faculty of Sport Science, University of Extremadura, 10003 Cáceres, Spain; alvaromurillo@unex.es (A.M.-G.); svillafaina@unex.es (S.V.); palomaroh@unex.es (P.R.-D.); ngusi@unex.es (N.G.); 2 Department of Psychology and Anthropology, University of Extremadura, 10003 Caceres, Spain

**Keywords:** fibromyalgia, dual task, MMSE, MoCA, TUG, cognitive

## Abstract

Fibromyalgia is a syndrome that is characterized by widespread pain; fatigue; stiffness; reduced physical fitness; sleep disturbances; psychological symptoms, such as anxiety and depression; and deficits in cognitive functions, such as attention, executive function, and verbal memory deficits. It is important to analyze the potentially different performance on the Mini-Mental State Examination (MMSE) and the Montreal Cognitive Assessment (MoCA) test in patients with fibromyalgia as well as examine the relationship of that performance with physical and cognitive performance. A total of 36 women with fibromyalgia participated in the study. Participants completed the MoCA test, the MMSE, and the TUG physical fitness test under dual-task conditions. The results obtained on cognitive tests were 28.19 (1.74) on the MMSE and 25.17 (2.79) on the MoCA. The participants’ performance on cognitive tests was significantly related to the results of the TUG dual-task test. In this way, cognitive performance on a dual-task test can be used to support the diagnosis of cognitive impairment in patients with fibromyalgia. The MoCA test may be a more sensitive cognitive screening tool than the MMSE for patients with fibromyalgia.

## 1. Introduction

Fibromyalgia is a syndrome that is characterized by widespread pain; fatigue; stiffness; reduced physical fitness; sleep disturbances, in particular insomnia [1]; psychological symptoms, such as anxiety and depression [2]; and deficits in cognitive functions, such as attention, executive function, and verbal memory deficits [1,3]. The global prevalence of fibromyalgia is 2.7%, being more prevalent in 50-year-old or older women [4].

A significant body of evidence indicates that cognitive activity is involved in natural motor activities such as walking [5], which is an activity of daily living. When we say cognitive activity, we mean that there is thought. Therefore, when walking, at the same time, we may be thinking of the destination to reach and on what we plan to do there. We may be calculating the distances between obstacles, such as cars or other pedestrians, or even the time we will take to reach the destination. The simultaneously execution of a motor task (such as walking) and a cognitive task (such as thinking) is called a dual task [6]. In this regard, activities of daily living often require the ability to simultaneously perform a cognitive and a motor task, that is, dual-task performance [7]. When an individual faces a dual task in daily life, successful execution of both of these tasks (i.e., the motor task (walking) and the cognitive task (thinking)) decreases because of dual-task interference [6]. This interference occurs because the individual has to divide their attention resources between both of the tasks. “Attention is the cognitive mechanism through which the information that is received by our senses is filtered and/or cognitive resources are assigned to particular elements of that information that are relevant to the observer” [8]. A decrease in the successful execution of a motor task usually leads to accidents, such as falls, especially in older people [5]. In experimental contexts, to replicate the situation in which an individual is walking and cognitive activity occurs, the cognitive motor dual task is usually used [6]. Within this paradigm, participants are asked to perform a cognitive and a motor task at the same time. 

Previous studies have found that patients with fibromyalgia usually show reduced dual-task performance in comparison to healthy counterparts [9,10]. Moreover, the ability to perform daily activities is conditioned by physical fitness [11] and the fear of falling [12]. Therefore, physical function is a relevant outcome in patients with fibromyalgia [13]. There are several physical fitness tests that measure subjects’ mobility. Among these tests, we emphasize the Timed Up and Go (TUG) test, which is a widely used instrument that evaluates mobility and the risk of falls in older adults [14,15]. In the TUG test, patients have to get up from a chair without using the arm rests. Then, they have to walk 3 m as quickly as possible without running, turn around, walk back to the chair, and sit down without using arm rests [16]. The TUG test provides a valid prognostic assessment to predict falls in elderly people [17], and it has been associated with fall history, indicating that older adults with slower TUG scores report a fall history more often than those in other categories (or compared to other instruments) [18]. TUG scores have been associated with executive function in 70-year-old or older participants, “indicating that a longer time on the TUG is associated with lower executive function performance”. These associations have been found specifically using the trail making test (which evaluates the components of executive function that represent complex visual scanning, speed, attention, and the ability to shift sets) and the Stroop word-color test (which evaluates components of executive function representing a person’s ability to deal with conflicting stimuli) [19]. In addition, the dual-task TUG test has also been shown to be an effective tool to determine the risk of falls and to better discriminate between fallers and nonfallers, even in a scenario where all standard tests and measures are insufficient to show significant differences [20].

A previous study evaluated the influence of dual-task conditions in patients with fibromyalgia in comparison to healthy people [9]. In this study, both groups performed three physical fitness tests (arm curl, handgrip, and 10-step stair tests) under two conditions: (a) regular condition (single task), that is, without performing any additional cognitive activity, and (b) dual-task condition, that is, while thinking of three words that were given before each test and had to be recalled and verbalized after the execution of each test. As a result, women with fibromyalgia showed lower physical performance (achieving the significance level in the arm curl test and in the 10-step stair test) than healthy people under both single- and dual-task conditions [9]. In addition, Moriarty et al. conducted a review of clinical and preclinical research to establish whether chronic pain negatively affects cognition. The authors concluded that pain is associated with impaired cognitive function and might be a consequence of competing limited neural resources, neuroplasticity, and/or dysregulated brain neurochemistry [21].

The Mini-Mental State Examination (MMSE) [22] is a widely used screening tool for dementia [23], and it is validated for Spanish-speaking communities [24]. A meta-analysis of the accuracy of the MMSE in the detection of dementia and mild cognitive impairment showed that the cut-off of 23/24 was the most used in the 34 analyzed studies [25]. However, the MMSE has a series of limitations, especially regarding its use in more educated patients [26]. To address this problem, the Montreal Cognitive Assessment (MoCA) was developed as a tool to screen patients with mild cognitive impairment whose performance on the MMSE is usually located at the normal range [27]. A meta-analysis compared the diagnostic accuracy of a range of cut-off scores from nine studies that evaluated the validity of the MoCA. The results showed that a cut-off score of 23/30 has a better diagnostic accuracy across parameters than the originally recommended 26/30 cut-off score, reducing the rate of false positives [28]. 

Previous research has examined which cognitive screening tool (MMSE vs. MoCA) is more effective [28,29]. In this regard, the MoCA seems superior to the MMSE in the detection of cognitive impairment in patients at a higher risk for incident dementia [29]. Another study supported that the MoCA is, as a cognitive screening tool, superior to the MMSE in detecting cognitive decline in its early stages [30]. In addition, the MoCA, but not the MMSE, has adequate psychometric properties as a screening instrument for the detection of mild cognitive impairment or dementia in Parkinson’s disease [31]. However, that question has not been studied in patients with fibromyalgia, although cognitive impairment is one of the symptoms [32]. 

Considering all the above, the aim of the present study was to analyze the differences between the cognitive assessment of both the MMSE and MoCA in patients with fibromyalgia as well as examine the relationship of the cognitive assessment score with physical and cognitive performance under dual-tasks conditions.

## 2. Experimental Section

### 2.1. Design

This study was designed as an explorative and descriptive study.

### 2.2. Participants

A total of 36 women with fibromyalgia participated in the study. The inclusion criteria were: women aged between 30 and 75 years old diagnosed with fibromyalgia by a rheumatologist, according to the criteria that have been established by the American College of Rheumatology [1]. Participants were excluded from the study if they met the following exclusion criteria: (a) being pregnant and (b) not being able to stand and sit on a chair once.

All participants provided signed written consent to participate in the study. All procedures were approved by the university’s bioethical committee (approval number: 62/2017) and followed the recommendations of the updated Declaration of Helsinki.

### 2.3. Procedures

First, anthropometric measurements of the participants were taken to calculate the body mass index (BMI). Subsequently, the participants completed the Spanish version of the Fibromyalgia Impact Questionnaire (FIQ), which evaluates the impact of symptoms of the disease from 0 to 100, indicating the minimum to maximum impact, respectively. The FIQ is an extensively validated fibromyalgia-specific tool that captures the overall effects of fibromyalgia symptomatology (pain, fatigue, feeling rested, stiffness, anxiety, depression, physical impairment, feel good, or work missed) [33,34,35]. After, trained research staff administered the MoCA and MMSE, both previously used in patients with fibromyalgia [36,37,38]. 

The MoCA test is a brief cognitive screening tool with high sensitivity and specificity for detecting mild cognitive impairment, which evaluates the following cognitive abilities: attention, concentration, executive functions (including the capacity for abstraction), memory, language, visuoconstructive abilities, calculation, and orientation [27]. The required administration time is approximately ten minutes. The maximum score is 30 [39]. In the present study, a score of 23 or higher was considered normal. In this sense, a meta-analysis compared the diagnostic accuracy of a range of cut-off scores of nine studies that evaluated the validity of the MoCA test. Its results showed that a cut-off score of 23/30 has a better diagnostic accuracy across parameters than the originally recommended 26/30 cut-off score, reducing the rate of false positives [28].

The MMSE is a widely used test of cognitive function; it includes tests of orientation, attention, memory, language, and visual-spatial skills. A higher score represents a better cognitive state. In the present study, a score of 24 or higher was considered normal. A meta-analysis of the accuracy of the MMSE in the detection of dementia and mild cognitive impairment showed that the cut-off of 23/24 was the most used in the 34 studies that were analyzed [25].

Finally, the participants completed the TUG physical fitness test under single- and dual-task conditions. The order of single- and dual-task conditions was randomized. Particularly, under the single-task condition, the participants had to get up from a chair without using arm rests; then, they had to walk 3 m as quickly as possible without running, turn around, walk back to the chair, and sit down without using arm rests [16]. The dual-task condition consisted of counting aloud backward two by two while performing the tests, starting from a random number higher than 100 [40].

### 2.4. Data Analysis

The SPSS statistical package version 24 (IBM Corp., Armonk, NY, USA) was used to analyze the data. Descriptive analyses were conducted to obtain means and standard deviations (SDs) regarding participants’ age and anthropometric measurements. Moreover, parametric and nonparametric tests were conducted based on the results of the Shapiro–Wilk test. Thus, Pearson´s and Spearman’s rho correlation analyses were conducted to evaluate the relationship between the participants’ performance on cognitive tests (MMSE vs. MoCA) and cognitive performance on the TUG test under dual-task conditions. In addition, the dual-task cost (DTC) of the TUG test [41] was calculated as follows:

DTC = (Dual-task TUG time—Single-task TUG time)/Single-task TUG time

The TUG cognitive performance variable was calculated by dividing the number of cognitive hits (correct answers in the subtractions) between the seconds spent on the dual-task test.

## 3. Results

Table 1 shows the participants’ descriptive characteristics in terms of means and SDs, including age 55.11 (8.74) years, BMI 28.30 (3.44) kg/m^2^, FIQ-100 53.50 (20.31), and medications: analgesics/relaxants (33.3%), hypotensive drugs (11.1%), antidepressants (41.7%), and others (80.6%).

Table 2 shows the results of the participants’ performance on the TUG test, in terms of means and SDs, under single-task, 7.55 (1.96) seconds, and dual-task, 8.20 (2.30) seconds, conditions. In addition, this table shows the dual-task cost on the TUG test: −0.08 (1.11) seconds. Table 2 shows the results regarding the participants’ performance on the TUG test, which is related to cognitive performance: 0.53 (0.28). The number of cognitive hits, 4.14 (2.09), and misses, 0.11 (0.38), in the subtractions on the dual-task TUG test are also reported in Table 2. Finally, the results obtained on cognitive tests (MMSE: 28.19 (1.74) and MoCA: 25.17 (2.79)) are also reported in Table 2. 

Table 3 shows the correlation analyses between the participants’ performance on the TUG test under single- and dual-task conditions and the participants’ performance on cognitive tests (MMSE and MoCA test) without finding a correlation in any of the tests. In addition, the correlation analyses between cognitive test results and the dual-task cost, cognitive performance, and hits and misses on the TUG test under the dual-task condition are reported in Table 3. As can be observed, the participants’ performance on cognitive tests was only significantly related to the results of the TUG dual-task cognitive performance test.

Finally, results of the participants’ performance on the MMSE and the MoCA are shown in Table 4 and Table 5, respectively, in terms of frequencies. Regarding Table 4 (performance on the MMSE), only 2.8% of the participants had cognitive impairment (with the cut-off point at 24) and 25% of the participants answered all the questions correctly. Regarding Table 5 (on performance on the MoCA), 16.7% of the participants had cognitive impairment (with the cut-off point at 23) and only 5.6% of the participants answered all the questions correctly. Thus, the results showed a ceiling effect, with 25.0% of the participants achieving a perfect score on the MMSE compared to 5.6% on the MoCA.

## 4. Discussion

This is the first study to analyze, compare, and correlate the results of two well-known cognitive assessment tests (MMSE and MoCA) with the physical and cognitive performance on the TUG test under single- and dual-task conditions in people with fibromyalgia. We found positive correlation between the results of the cognitive tests (MMSE and MoCA) and the cognitive performance on the TUG test under dual-task condition. According to the results of the MMSE, only 2.8% of the participants had cognitive impairment. However, according to the results of the MoCA test, 16.7% of the participants had cognitive impairment. In both tests, the strictest cut-off points were used to avoid false positives (23 in the MoCA and 24 in the MMSE). In addition, although less-strict cut-off points were used in each test (26 in the MoCA and 27 in the MMSE), a difference between the results of both tests still existed. Finally, using less strict cut-off points in each test, 13.9% of the population had cognitive impairment on the MMSE and 55.6% on the MoCA.

Despite the experimental results of neuroimaging studies, a diagnosis of cognitive and behavioral impairment in patients with fibromyalgia still relies on the use of validated tests of neuropsychiatric assessment [42]. In addition, several studies have confirmed that a diagnosis of cognitive impairment using a single test is not enough and have recommended relying on other methods to ensure the diagnosis [31,43,44]. Furthermore, in people with fibromyalgia, comorbid symptoms such as depression, anxiety, insomnia, and fatigue may impact cognitive function, but they do not entirely explain the mental impairment. Chronic pain may disrupt attention and induce neuroplasticity in the central nervous system [42]. Thus, it is difficult to find a gold standard for the evaluation of cognitive impairment in fibromyalgia. Therefore, it is important to know which of the cognitive tests used is most adequate to help in the diagnosis of cognitive impairment in fibromyalgia.

The findings of the present study are consistent with most of the research that has been conducted to date: the MMSE does not perform well as a screening instrument (mainly due to the ceiling effect). This may be partially due to a lack of sensitivity to milder cognitive deficits [31,45]. The present results show the instrument ceiling effect, with 25.0% of the participants achieving a perfect score on the MMSE compared to 5.6% on the MoCA. As shown in a previous study, the poorer performance of the MMSE in detecting cognitive impairment may be due to a series of several factors, apart from a lack of sensitivity to milder cognitive deficits. First, the MMSE may be less effective at assessing complex cognitive domains such as visuospatial skills, executive function, and abstract reasoning. In addition, the MMSE includes one test for attention, while the MoCA includes two additional tests (digit span and vigilance). Similarly, the three-item test on delayed recall in the MMSE is less difficult than the five-item test on delayed recall in the MoCA. Thus, the attention and delayed-recall items in the MMSE are easier than in the MoCA [46]. The differences that have been described above between performance on the MMSE and performance on the MoCA are important when assessing cognitive impairment in patients with fibromyalgia because a previous study found memory and vocabulary deficits in patients with fibromyalgia, showing that memory function in patients with fibromyalgia is not age-appropriate [47]. For the above reasons, it is possible to recommend the MoCA test to help in the diagnosis of mild cognitive impairment in fibromyalgia patients.

The results obtained in the TUG test are similar to results of other studies on fibromyalgia patients, both under single-task [40,48,49] and dual-task [40] conditions. Among the measures that were obtained from the dual-task TUG test, we identified cognitive performance. This variable was calculated by dividing the number of cognitive hits between the seconds spent on the dual-task test. In this regard, we found a positive correlation between the results of both cognitive tests (MMSE and MoCA) and the cognitive performance on the TUG test under a dual-task condition. A previous study found significant correlations between performance on the TUG test (under dual-task conditions) and MoCA and MMSE scores [41]. In this study, the cognitive task consisted of reciting the days of the week in reverse order starting from Sunday while performing the TUG test. These findings suggested that the TUG test under the dual-task condition might be used in clinical practice as a functional and practical test for the early screening of cognitive dysfunction among older adults. However, the authors recommended additional studies including more challenging cognitive tasks during the TUG test to obtain stronger correlations with cognitive tests. Therefore, in the current study, we selected a dual-task condition that consisted of counting aloud backward two by two while performing the tests, starting from a random number higher than 100 [40]. This might be the reason we found a correlation only between cognitive performance on the TUG test under the dual-task condition and the results of both cognitive tests (MMSE and MoCA), being even stricter for the assessment of cognitive impairment. Moreover, the cognitive task used under the dual-task condition (counting aloud backward two by two) requires some of the cognitive domains that are assessed on the MMSE or the MoCA, such as working memory, calculation, or attention, among others. This is relevant since when clinicians or researchers conduct evaluations under the dual-task paradigm, physical performance is usually presented, ignoring cognitive performance under the dual-task condition. However, our results suggested that cognitive performance under the dual-task condition provides us with cognitive information in the same line using the MoCA and MMSE. Thus, to complement the patients’ cognitive assessments, this variable should be considered rather than physical performance under the dual-task condition or even the DTC.

Previously, it was shown that physical performance on the TUG test under single- and dual-task conditions could predict mild cognitive impairment [19]. However, the results of the current study did not show correlations between physical performance on the TUG (single- and dual-task) test and cognitive performance on cognitive tests. Importantly, we found a positive correlation between the results of cognitive tests (MMSE and MoCA) and cognitive performance on the TUG test under the dual-task condition. This relationship between cognitive performance on cognitive tests and cognitive performance on the TUG test under the dual-task condition may help support the cognitive impairment diagnosis in fibromyalgia patients, highlighting its importance.

The present study had limitations: the small number of participants and all the participants being women. Thus, the results cannot be extrapolated to men with fibromyalgia. Moreover, our data cannot be compared with normative data (for the MoCA and the MMSE) due to both the specificity of the fibromyalgia population and the lack of normative values for this population. Furthermore, since there is no gold standard to measure cognitive impairment in patients with fibromyalgia, the sensitivity and specificity of both cognitive tests could not be calculated in the present study. The main conclusion was based on the ceiling effect of both tests.

## 5. Conclusions

The MoCA may be a more sensitive cognitive screening tool than the MMSE for patients with fibromyalgia. The results of both cognitive tests correlated with cognitive performance on the dual-task TUG test. As such, cognitive performance on a dual-task test can be used to support the diagnosis of cognitive impairment in patients with fibromyalgia because it provides a functional assessment related to real-life activities, although more research is needed to generalize the present results to populations with fibromyalgia. 

## Figures and Tables

**Table 1:** Characteristics of the participants.

**Variable (n = 36) (Maximum–Minimum Values)**	**Mean (SD)**
Age (years) (34–70)	55.11 (8.74)
BMI (kg/m^2^) (24–39)	28.30 (3.44)
FIQ-100 (9–86)	53.50 (20.31)
**Medication**	**n (Percentage)**
Analgesics/Relaxants	12 (33.3%)
Hypotensive drugs	4 (11.1%)
Antidepressants	15 (41.7%)
Others	29 (80.6%)

BMI, body mass index; FIQ, Fibromyalgia Impact Questionnaire; SD, standard deviation.

**Table 2:** Performance on the TUG test, dual-task TUG test, and cognitive tests (MMSE and MoCA).

Variable (n = 36) (Maximum–Minimum Values)	Mean	SD
TUG (5.43–15.06)	7.55	1.96
TUG Dual-Task (5.39–15.42)	8.20	2.30
TUG Dual-Task Cost (−0.07–0.45)	0.08	0.11
TUG Cognitive Performance (0–1)	0.53	0.28
TUG Hits (0–7)	4.14	2.09
TUG Misses (0–2)	0.11	0.38
MMSE (23–30)	28.19	1.74
MoCA (19–31)	25.17	2.79

TUG, Timed Up and Go; MMSE, Mini-Mental State Examination; MoCA, Montreal Cognitive Assessment.

**Table 3:** Correlations between the TUG test and cognitive tests.

Variable (n = 36)	MMSE		MoCA Test	
TUG	Spearman’s CC	−0.206	Spearman’s CC	−0.136
	*p*-value	0.227	*p*-value	0.082
TUG Dual-Task	Spearman’s CC	−0.260	Spearman’s CC	−0.105
	*p*-value	0.126	*p*-value	0.541
TUG Dual-Task Cost	Spearman’s CC	−0.077	Spearman’s CC	0.020
	*p*-value	0.657	*p*-value	0.906
TUG Dual-Task Cognitive Performance	Spearman´s CC	0.355	Pearson´s CC	0.348
	*p*-value	0.034 *	*p*-value	0.038 *
TUG Hits	Spearman’s CC	0.169	Spearman’s CC	0.297
	*p*-value	0.324	*p*-value	0.078
TUG Misses	Spearman’s CC	0.059	Spearman’s CC	−0.187
	*p*-value	0.732	*p*-value	0.275

* *p*-value <0.05. CC, correlation coefficient; TUG, Timed Up and Go; MMSE, Mini-Mental State Examination; MoCA, Montreal Cognitive Assessment.

**Table 4:** Results regarding the participants’ performance on the MMSE.

MMSE (n = 36)	Frequency	Percentage	Accumulated Percentage
0–22	0	0%	0
23	1	2.8%	2.8%
24	0	0%	2.8%
25	3	8.3%	11.1%
26	1	2.8%	13.9%
27	5	13.9%	27.8%
28	7	19.4%	47.2%
29	10	27.8%	75.0%
30	9	25.0%	100%
Total	36	100%	

**Table 5:** Results regarding the participants’ performance on the MoCA test.

MoCA (n = 36)	Frequency	Percentage	Accumulated Percentage
0–19	1	2.8%	2.8%
20	2	5.6%	8.3%
21	1	2.8%	11.1%
22	2	5.6%	16.7%
23	4	11.1%	27.8%
24	2	5.6%	33.3%
25	8	22.2%	55.6%
26	2	5.6%	61.1%
27	7	19. 4%	80.6%
28	5	13.9%	94.4%
29	0	0%	94.4%
30	2	5.6%	100%
Total	36	100	

## Data Availability

All data are available by the corresponding author (S.G.) upon reasonable request.
